# Mobility related physical and functional losses due to aging and disease - a motivation for lower limb exoskeletons

**DOI:** 10.1186/s12984-018-0458-8

**Published:** 2019-01-03

**Authors:** Martin Grimmer, Robert Riener, Conor James Walsh, André Seyfarth

**Affiliations:** 1 Lauflabor Locomotion Lab, Technische Universität Darmstadt, Magdalenenstr. 27, Darmstadt, 64289 Germany; 2 Sensory-Motor Systems (SMS) Lab, Institute of Robotics and Intelligent Systems (IRIS), Department of Health Sciences and Technology, ETH Zurich, Tannenstr. 1, Zurich, 8092 Switzerland; 3 Harvard Biodesign Lab, John A. Paulson School of Engineering and Applied Sciences, Wyss Institute for Biologically Inspired Engineering, Harvard University, 60 Oxford Street, Cambridge, 02138 MA United States

**Keywords:** Exoskeleton, Assistance, Aging, Walking, Mobility, Impaired, Motivation

## Abstract

**Background:**

Physical and functional losses due to aging and diseases decrease human mobility, independence, and quality of life. This study is aimed at summarizing and quantifying these losses in order to motivate solutions to overcome them with a special focus on the possibilities by using lower limb exoskeletons.

**Methods:**

A narrative literature review was performed to determine a broad range of mobility-related physical and functional measures that are affected by aging and selected cardiovascular, respiratory, musculoskeletal, and neurological diseases.

**Results:**

The study identified that decreases in limb maximum muscle force and power (33% and 49%, respectively, 25–75 yrs) and in maximum oxygen consumption (40%, 20–80 yrs) occur for older adults compared to young adults. Reaction times more than double (18–90 yrs) and losses in the visual, vestibular, and somatosensory systems were reported. Additionally, we found decreases in steps per day (75%, 60–85 yrs), maximum walking speed (24% 25–75 yrs), and maximum six-minute and self-selected walking speed (38% and 21%, respectively, 20–85 yrs), while we found increases in the number of falls relative to the number of steps per day (800%), injuries due to falls (472%, 30–90 yrs) and deaths caused by fall (4000%, 65–90 yrs). Measures were identified to be worse for individuals with impaired mobility. Additional detrimental effects identified for them were the loss of upright standing and locomotion, freezing in movement, joint stress, pain, and changes in gait patterns.

**Discussion:**

This review shows that aging and chronic conditions result in wide-ranging losses in physical and sensory capabilities. While the impact of these losses are relatively modest for level walking, they become limiting during more demanding tasks such as walking on inclined ground, climbing stairs, or walking over longer periods, and especially when coupled with a debilitating disease. As the physical and functional parameters are closely related, we believe that lost functional capabilities can be indirectly improved by training of the physical capabilities. However, assistive devices can supplement the lost functional capabilities directly by compensating for losses with propulsion, weight support, and balance support.

**Conclusions:**

Exoskeletons are a new generation of assistive devices that have the potential to provide both, training capabilities and functional compensation, to enhance human mobility.

## Introduction

Improving quality of life is a goal of modern society. Quality of life studies assess the physical condition, as poor physical condition can limit daily mobility and the ability to move and work. One of the main causes of limitations in daily mobility might be the physical losses that occur with increasing age, which results in reduced muscle force or muscle power. These losses reduce the functional capacity, including both ability and intensity, for movement tasks such as level walking or climbing stairs.

Many secondary problems are related to the physical and functional capacity. A greater number of steps per day is associated with metrics that are indicative of positive health, such as blood pressure [1], diabetes related glucose tolerance [2], body mass index [3], risk of cardiovascular disease [4], risk of coronary heart disease [4], lipid profiles [4] and mortality [5]. Changing posture can also help to reduce secondary medical symptoms like bladder infections, stomach problems, pressure sores, respiratory problems, fatigue, bowel problems, and osteoporosis [6].

Maintaining or improving the physical condition is of critical importance as our population ages. The World Health Organisation (WHO) estimated an increase in the number of older adults above the age of 65 years from 524 million in 2010 to 1.5 billion in 2050, which is an increase from 8% to 16% of the world’s population [7]. In addition to age-related degenerations, a larger proportion of the population is expected to be affected by mobility-related impairments due to chronic diseases. Worldwide more than 500 million people suffer from a permanent reduction of the physical and functional capacity due to diseases affecting the respiratory, cardiovascular, musculoskeletal or neurological systems (Table 1). Concerning the cardiovascular system specifically, it is predicted that there will be a large increase of cases until 2040 [8].

**Table 1:** Diseases analyzed in this work with an influence on the mobility related physical and functional capacity and their worldwide prevalence

System	Disease	Prevalence worldwide in million	Source
Respiratory	Chronic obstructive pulmonary disease (COPD)	64-330	[172, 173]
	Cystic fibrosis (CF)	0.1	Estimated based on [174]
Cardiovascular	Coronary artery disease (CAD)	93	[173]
	Peripheral vascular disease (PVD)	202	[175]
Musculoskeletal	Osteoarthritis (OA)	151	[172]
	Facioscapulohumeral muscular dystrophy (FSHD)	0.87	Estimated based on [176]
Neurological	Parkinsons disease (PD)	5.2	[172]
	Cerebral palsy (CP)	16	Estimated based on [177]
	(incomplete) Spinal cord injury ((i)SCI)	3.5	Estimated based on [178]

Short descriptions and information on the prevalence estimations are provided in the Appendix

For individuals with impaired mobility, the reduced mobility is more prevalent than other aspects of life such as employment or education [9]. In a study on determinants that increase health-related quality of life for people with Chronic obstructive pulmonary disease (COPD), an improvement in physical performance was identified as a primary contributor [10]. Walking and standing were the mobility functions that were most desired for people with spinal cord injury (SCI) [11].

The increasing population of elderly individuals and individuals with disease-related impaired mobility suggest that there is a need for mobility solutions to secure an independent daily life.

### Wearable robotics for locomotion assistance

To date, mostly passive systems are used to support mobility and independence. To assist with walking, crutches or walkers are used, as they are able to unload joints to avoid pain caused by musculoskeletal diseases such as osteoarthritis. Braces are used to stabilize joints. In addition, these devices can help to improve balance, which is limited due to muscle strength, endurance, or neurological reasons. However, the functional user benefits are limited for passive devices such as ankle-foot orthoses [12]. In order to provide increased capabilities, powered devices such as electrical wheelchairs replace their passive counterparts for those with limited or no walking ability.

**Fig. 1:**
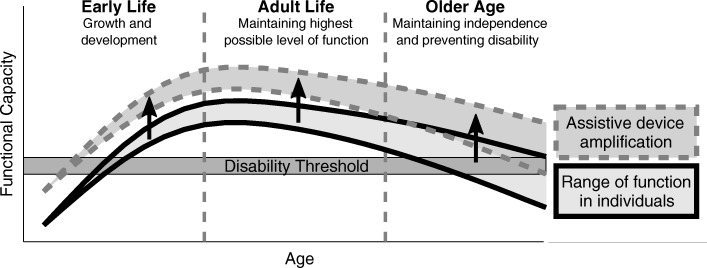
Functional capacity over the course of life. Changes in the environment can lower the disability threshold. Assistive devices provide the potential to increase the level of function for all age groups. Thus, fewer individuals would fall below the disability threshold for certain capabilities (modified from [165])

Exoskeletons are a new generation of powered technical aids to address physical and functional deficits. Furthermore, an augmentation (e.g. walking with less effort) of the physical and functional capacity is possible (Fig. 1). Upper and lower limb exoskeletons have been designed for assisting with lifting heavy objects (HAL, [13]), sustained and fatigue-free load carrying (HULC [14], BLEEX [15], Harvard Exosuit [16]), and manufacturing (Honda Assist [17]) or medical applications (Nursing assist suit, [18]). In the medical field, stationary and autonomous rehabilitation systems have been developed to assist patients in regaining walking ability after injury. Examples for stationary devices are the Lokomat [19], Lopez [20] or G-EO [21]. Examples for autonomous systems to assist the hip and the knee are the HAL [22], the Ekso [23], the REX [24], the ReWalk [25] or the Indego [26] exoskeleton.

In addition to these developments, minimalistic exoskeletons have been developed that assist single joints or that use single actuators to assist multiple joints. Tethered minimalistic systems to assist the ankle are the motor-based exoskeleton emulator from Carnegie Mellon University [27] and the pneumatic ankle exoskeleton from the University of Michigan [28]. Multiarticular actuation has been used for tethered [29] and autonomous [16] versions of the Harvard exosuit, and for the autonomous Myosuit [30]. Autonomous examples of minimalistic systems that address single joints are the ankle exosuit from Harvard [31], the ankle exoskeleton from MIT [32], or the hip exoskeletons from Samsung [33], Honda [34], Georgia Tech [35], and Sant’Anna [36]. While tethered systems have been used for rehabilitation and research, autonomous systems allow for the assistance with walking or to provide walking capability (exchange of wheelchair) in daily life.

### Study focus

This narrative review aimed to summarize and quantify losses in mobility-related physical and functional parameters over the course of the human adult lifespan that could potentially be addressed with wearable robotics. Additionally, selected diseases involving the cardiovascular, respiratory, musculoskeletal, and neurological systems were analyzed to determine if affected people suffer from greater mobility-related losses compared to the effects due to aging. Finally, we summarized the physical requirements to perform the daily life tasks of level walking, inclined walking, and climbing stairs.

In the discussion we confronted both, losses and daily movement requirements, to provide an understanding for mobility limitations of the analyzed populations. Further it was discussed how the functional capacity can be improved with a special focus on possibilities with the help of exoskeletons. Although our aim was not to summarize different exoskeleton solutions or control approaches to overcome the identified functional losses, we provided a short perspective based on previously published work.

## Methods

### Selection of physical and functional parameters

The selection of the physical parameters was based on representative values for humans to perform work over short durations (muscle force, muscle power) and prolonged durations (VO_2_max). Functional parameters were selected to quantify effects on daily performance. As 20% of all daily trips for adults are performed by walking [37], we selected steps per day and walking speed as indicators for changes in this most basic mobility function. As balance is a key function for sustained upright standing and locomotion, balance quality was assessed using surrogate measures of falls, including the number of fall injuries and the number of deaths caused by falls. Injuries and deaths were added to have a measure for the relevance of fall prevention and treatment. Upper and lower limb reaction times were included to identify a possible source for changing amount of falls with increasing age. As physical deficits are not the only source for falls, additional changes in the sensory systems of humans were summarized.

### Selection of mobility related diseases

The selection of mobility-related diseases (Table 1) was made based on different classes of diseases used in previous work to predict changes for hospital admissions and costs [8]. From this study, four classes of diseases were selected: respiratory, cardiovascular, musculoskeletal, and neurological. From each class, representative diseases were selected. Some diseases were selected based on a list of diseases mentioned as factors with a detrimental influence in the six-minute walk test [38]. Additionally, diseases were selected for which exoskeletons are currently used, or where the authors see a potential for exoskeleton assistance. The intention of the selection was to show the broad range of mobility-related diseases rather than to provide a complete overview that includes all possible diseases.

The selected diseases, including their abbreviations and their worldwide prevalence, can be found in Table 1. The selected respiratory diseases were Chronic obstructive pulmonary disease (COPD) and Cystic fibrosis (CF). Cardiovascular diseases include Coronary artery disease (CAD) and Peripheral vascular disease (PVD). Selected musculoskeletal diseases were Osteoarthritis (OA) and Facioscapulohumeral muscular dystrophy (FSHD). Representatives for neurological diseases included Parkinsons disease (PD), Cerebral palsy (CP), and (incomplete) Spinal cord injury ((i)SCI) were selected. Worldwide prevalence numbers were cited from the literature, although some were estimated based on literature. Further descriptions on the diseases and the prevalence estimations can be found in the Appendix.

### Literature search

The literature search was performed using Google Scholar. Search terms included the names of the physical and functional parameters as well as the names or abbreviations of the mobility-related diseases. These search terms were combined with the terms: *walking, muscle, torque, human, oxygen, VO2max, age, aging, elderly, adult, speed, velocity, balance, test, reason, cause,* or *gait*. To find or estimate the worldwide statistics of cases for each selected disease the search terms *prevalence, incidence,* and *worldwide* were included. Partially, the worldwide statistics of cases was identified using sources of the World Health Organization (WHO) identified using the search term *World Health Organization* in combination with the previously mentioned terms in Google. In addition to the direct literature search, the electronic searches were supplemented by reviewing the retrieved articles for relevant content and references regarding this content.

## Results

The “Results” section consists of two major subsections. In the first subsection, aging- and disease-related losses in physical and functional parameters, and the reasons for the losses, are summarized. The second subsection summarizes differences in physical parameter requirements regarding daily locomotion tasks such as level walking or stair climbing.

The parameter changes with increasing age (in percent) and the mean age values for the compared groups are provided (e.g. 25 to 75 yrs).

### Losses in physical and functional parameters

#### Maximum muscle force and power

**Fig. 2:**
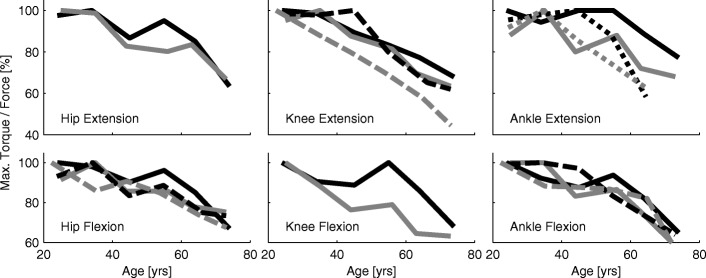
Torque and force development. Maximum torque and maximum force development for the hip, the knee, and the ankle extension and flexion with increasing age. Solid lines contain data published by Harbo et al. [39] (178 subjects, 15 to 83 yrs, isokinetic peak torque). Dashed lines contain data of Bohannon [40] (231 subjects, 20 to 79 yrs, hand held dynamometer peak force). Dotted lines contain data from Fugl-Meyer et al. [41] (135 subjects, 20 to 65 yrs, isokinetic peak torque). Black lines are for male, gray lines for female subject data

**Fig. 3:**
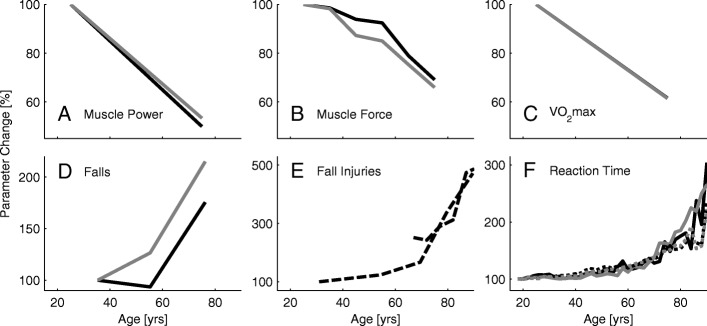
Summary of age related parameters. Changes with age in maximum muscle power (**a**), maximum muscle force (**b**), maximum oxygen consumption (**c**), self reported falls (**d**), injuries due to falls (**e**), and reaction time (**f**). Black lines represent male, gray lines female and dashed lines mixed groups. **a** Muscle power data was assessed by jumping mechanography (89 male, 169 female, 18-88 yrs) [46]. **b** Muscle force data is the mean of the curves presented in Fig. 2. **c** Maximum oxygen consumption was assessed in treadmill walking from (619 male, 497 female, 18-94 yrs) [54]. The relation of VO_2_max and age is described as *y*=51.23−0.33·*x* for males and *y*=41.74−0.27·*x* for females. **d** Changes in self reported falls (one minimum in the last two years) for three age groups in percent. Age means were 35.3 (20–45, *n*=292), 55.3 (46–65, *n*=616), and 76.2 (>65, *n*=589) years. The relative amount of male fallers is 16.8, 15.7, and 29.5 percent and of female fallers is 20, 25.3, and 43 percent with increasing age [118]. **e** Increases of injuries due to falls (survey, 30–90 yrs) for the Canadian (dashed, [123]) and the US (solid, [124]) population with 100% set for 30 years old of [124]. Absolute values are about 20 to 100 falls with injury per 1000 population for the 30 and 90 years old respectively. **f** Relative change with age (100% at 18 yrs) of single (dotted) and choice (solid) reaction time of 7130 subjects (18-90 yrs, [103]). Absolute values range from 287 ms to 872 ms for the single and 567 ms to 1129 ms for the choice reaction. Data was acquired using a single button that had to be pressed when showing a number in a display. Choice reaction time included pressing one out of four different buttons

Maximum lower limb torques and forces decrease with increasing age for the hip, knee, and ankle extensors and flexors ([39–41], Fig. 2). Mean values for all lower limb muscle groups (flexors and extensors of the hip, knee, and ankle) show a decrease from the age of 25 to the age of 75 of 31% and 34% for males and females, respectively, which is a decrease of eight percent per decade (Fig. 3b).

Further decreases compared to healthy subjects were reported for the lower extremity of people with respiratory (COPD), cardiovascular (PVD [42]), musculoskeletal (FSHD [43], OA [44]), and neurological (CP [45]) diseases.

Lower limb extensor power reductions (25 to 75 yrs) were almost equal for males and females (50% and 47%, respectively) with a decrease by approximately 13% each decade as evaluated using a jumping test (Fig. 3a, [46]). When comparing lower limb muscle force, maximum oxygen consumption, and lower limb muscle power, muscle power had the strongest correlation to self-reported functional status in older adults [47].

As studies have demonstrated further reductions in maximum muscle forces for respiratory, cardiovascular, muscoloskeletal, and neurological diseases [42, 43, 45, 48], it is expected that maximum muscle power is decreasing for these diseases as well. Studies that have evaluated maximum muscle power found reductions for individuals with COPD [49], CP [50], and OA [51] compared to the healthy reference groups.

Studies have shown that reasons for the losses in muscle force and power are due to changes in muscle function, architecture, and mass, however, changing tendon properties and body composition may also contribute [46]. One of the most important causes for the decline in muscle mass and function is physical inactivity [52]. Next to inactivity, many other muscle-related and non-muscle-related factors, such as hormones, probably cause the decline [53].

#### Maximum oxygen consumption

**Fig. 4:**
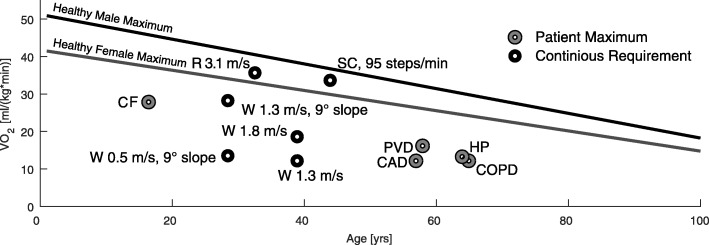
Oxygen consumption in relation to age and for different activities and diseases. VO_2_max decreases for healthy males (black line) and healthy females (gray line) with age. Example requirements of continuous level and incline walking (W, [149, 150]), running (R, [151]), and climbing stairs [152] are indicated by a black circle. VO_2_max values for people with peripheral vascular disease (PVD, [60]), coronary artery disease (CAD, [58]), chronic obstructive pulmonary disease (COPD, [56]), and cystic fibrosis (CF, [57]) and hemiparesis (HP, [50]) are indicated by a gray circle. Age related trends for both genders are from linear fits of 619 males and 497 females with an age between 18 to 95 years [14]

Maximum oxygen consumption (VO_2_max) was seen to be reduced by approximately 58% when comparing 20 years old with 80 years old subjects (45 to 26 ml ·kg^−1^·min^−1^, respectively) with a decrease of 10% each decade [54]. An analysis based on other datasets found similar magnitudes [55]. While the absolute values for males were higher than for females (Fig. 4), the relative decrease with age was similar (Fig. 3c).

**Fig. 5:**
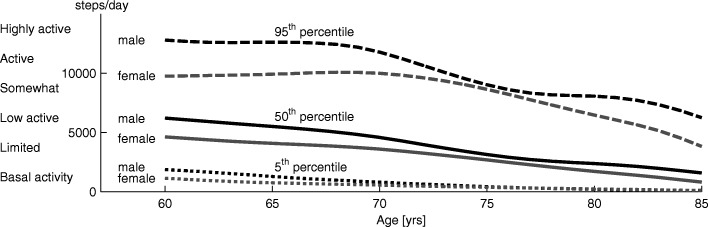
Steps per day. Percentiles of steps per day for males (black) and females (gray) from the age of 60 to 85 years. Five percent of the population achieves less than the 5^*th*^ percentile (dotted line) of steps per day, 50% is below the 50^*th*^ percentile (solid), and 5% is above the 95^*th*^ percentile (dashed). Data was taken from a US study [87] including results of 1196 60+ year old participants

Additional reductions in VO_2_max were found for the respiratory (COPD [56], CF [57]) and cardiovascular (CAD [58, 59], PVD [60]) diseases (Fig. 5). For young adults with FSHD, VO_2_max was found to be slightly lower than the healthy average [61]. No differences in VO_2_max were found between a healthy reference group and individuals with Parkinson’s disease [62].

The decrease in VO2max are primarily related to reductions in maximum heart rate and lean body mass [55]. While physical training is not able to influence the maximum heart rate, it can reduce the decrease in lean body mass [55].

#### Walking speed

A summary of 27 studies (Fig. 5) identified a self-selected level walking speed of approximately 1.35 m/s for young adults (20 yrs). Up to the age of 85, a decrease to 1.07 m/s was identified (21%) with most of the loss occurring between 60 and 85 (18% decrease starting at 1.3 m/s). Bohannon [63] identified similar trends and also showed that the maximum walking speed of adults decreases for males and females from 2.5 m/s to 1.9 m/s, a reduction of 24%. Based on [64], six-minute maximum walking speed was found to decrease from 2.1 m/s to 1.3 m/s between the age of 20 and 85 years (38% reduction).

In [65] it was found that, similar to level walking, uphill and downhill walking speed decrease with age. Uphill walking resulted in greater reductions in walking speed for the older adults (55–75 yrs) than for younger subjects (10–55 yrs). Similar to uphill walking, stair climbing speed (cadence) decreases in the older adults [66, 67].

Further reductions in walking speed were identified for almost all analyzed diseases. Six-minute walking speed decrease for people with COPD [68], PVD [69], and CAD [58, 59] (Fig. 5). People with mild to moderate CF were able to walk as fast as healthy subjects in the six-minute walking test but experienced a significant decrease in oxygen saturation and increased breathlessness perception during exercise [70]. A reduced walking speed, compared to the healthy reference group, was also identified for people with FSHD (Fig. 5, [71]) and OA [72]. In a group of young adults with CP, six-minute walking velocity was reduced compared to healthy (range: 0.25 to 1.7 m/s, Fig. 5, [73]). Reduced walking speeds were also found for people with PD [74] and iSCI [75]. The distance that individuals with iSCI walked in six minutes varied between 23 and 475 m.

Muscle strength and pain were identified as some of the reasons for reduced walking speed with increasing age [76, 77]. In treadmill walking (0.8 m/s), increased energy expenditure (29%) with age was identified when comparing women with a mean age of 42 ±1 years to a group of woman with a mean age of 72 ±4 years [78]. Only a portion of this effect was due to an increase in body weight (approximately 3 kg difference). The other portion of the increase in walking energy expenditure may be due to decreased walking efficiency [79] or balance-related issues [78]. Additional possible reasons for reductions in walking speed were sensory losses, balance-related issues [80, 81], and fear of falling [82].

#### Steps per day

**Fig. 6:**
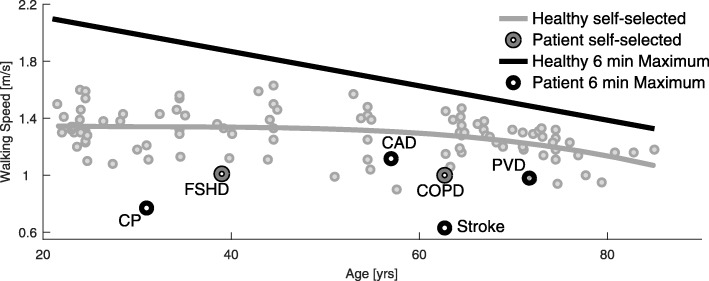
Walking speed, age and diseases. Self-selected (gray line) and six-minute maximum walking speed (black line) in relation to age for healthy subjects and examples of populations with diseases. Age-related self-selected speed data (small gray circles) was extracted from 27 studies including 100 data points of speed and age (see Appendix Table 2 for details). A trend was illustrated using polynomial curve fitting. The six-minute walking speed was measured with the six-minute walking test where subjects were encouraged to achieve the maximum distance by walking as fast as possible. The curve is based on the equation derived by [64] (40-80 yrs, *n*=155) in combination with input values that represent mixed gender groups (1.72m, 72kg). Patient data represents self-selected walking speed (dark gray circle) for patients with FSHD [71] and very serve COPD [166]. Due to limited availability of self-selected speed data, for CP [73], CAD [58], PVD [69], and stroke [167] walking speed (self-selected) for the six-minute walking test is shown. The healthy self-selected speed has a polynomial of order 3: *y*=−0.00000176·*x*^3^+0.00017·*x*^2^−0.00576·*x*+1.408

In total, adults walk between 6000 and 13,000 steps per day [83]. Physical and functional limitations result in decreased walking distance for older adults [84, 85]. Tudor-Locke and Basset [83, 86] classified steps per day into groups ranging from less than 2500 to above 12,500 steps per day (Fig. 6). Almost 50% of older adults above the age of 65 years belong to the classification “limited and basal activity” taking fewer than 5000 steps per day [87]. From the age of 60 years to the age of 85 years, the mean number of steps per day decreases by 71% to 80% (50th percentile of males and females). Active older adults have a 55% reduction in the number of steps per day over the same time period (Fig. 6). The number of steps taken per day are highly related to the neighborhood walkability rating and the number of reachable destinations (by maximum 20 min walking). The number of steps per day for a group of 74 ±4 (mean) year old females ranged from less than 3000 to more than 6000 for poor to excellent neighborhood conditions [88].

Respiratory, cardiovascular, muscoloskeletal, and neurological diseases showed further reductions in steps per day. For people with COPD, walking time decreased to almost half, standing time decreased to 66%, whereas sitting time and lying time were increased compared to an unaffected reference group [89]. Individuals with COPD were found [90] to take between 2140 (mean 66 ±10 yrs, [91]) and 3716 (mean 70 ±8 yrs, [92]) steps per day. For people with PVD, a significant decline in walking endurance was identified [93]. 4156 steps per day (mean 70 ±2 yrs,) were identified as a mean by Crowther et al. [94]. Steps per day were also reduced for individuals with OA [72]. For people with PD, a range from 7636 (mean 67 ±8 yrs) to 8756 (mean 71 ±11 yrs) was identified [90], which is above the mean of this age group (Fig. 6). On the other hand, people with spinal cord injury may not have locomotion capabilities at all. The impairment scale of the American Spinal Injury Association classifies SCI to four grades, ranging from no sensory and motor function in the sacral segments (grade A, 45%) to full range of motion and the ability to move against gravity with at least half of the key muscles (grade D, 30%, [95]). Depending on grade, people with incomplete SCI are able to stand up and walk. Abilities are clearly limited for most of them [96] and effort (cost of transport determined by metabolic cost of walking) was shown to be greater than double compared to unaffected reference subjects [97]. For mobility, most people with SCI require the use of a powered or manual wheelchair [98, 99]. For those with walking capabilities, steps per day ranged from 68 to 4468 (mean 42 ±13 yrs) with a mean of 1640 [99].

Multiple sources may contribute to the reduction in steps per day. In addition to retirement, which removes the necessity to travel to work, the reduction may be a result of physical reasons. Reasons for mobility impaired include reduced activity ([100], COPD), breathlessness ([70], CF), fatigue ([93], PVD), deoxygenation with calf pain ([101], PVD), pain ([102], OA), or increased effort ([96], incomplete SCI). As walking speed decreases, the number of reachable destinations (in 20 min, [88]) decreases. Thus alternatives modes of transportation, such as public transport, might be used and which might further decrease the steps per day.

#### Reaction time

Reaction time might be a key element in avoiding falls. It was shown that reaction time for the upper [103] and the lower extremities [104, 105] increased with age. For the upper extremity, it was demonstrated that this process seems to accelerate for people older than 65 years (Fig. 3f, [103]). If the fall recovery includes voluntary movements, choice reaction time (more than one option) might be more important than single reaction time. For both upper and lower extremities choice reaction time was shown to be greater than single reaction time [103, 105]. While single reaction time can more than double, choice reaction time can almost triple with increasing age (25 to 90 yrs, Fig. 3f, [103]).

Studies on further reductions in reaction time due to diseases were found for all respiratory, cardiovascular, musculoskeletal, and neurological diseases. Increases were found for COPD [106], OA [107], PD [108], CP [109], and incomplete SCI [110]. Choice reaction time was found to be an important risk factor for deaths from cardiovascular disease [111]. Subjects with evidence of cardiac or PVD have a significant reduction in cognitive function (including choice reaction time), which is equivalent to five years of aging [112].

Researchers hypothesized that the loss in reaction times is related to the maximum response execution speed rather than the sensory or motor programming processes involved in response initiation [104]. Other explanations include loss of maximum processing speed, processing robustness, and fluid intelligence with age [113]. Furthermore, it is assumed that older adults select a safer movement strategy with slower weight transference [105].

#### Balance and falls

Three major sensory systems are involved in enabling humans to maintain balance [114]. The visual system is required for path planning. The vestibular system senses linear and angular accelerations. The somatosensory system senses the velocity and the position of body segments, provides object contact information, and orientation of gravity. For all of them functional losses were identified with increasing age. Age-related decreases in vision were identified for visual processing speed, light sensitivity, dynamic vision, near vision and visual search [115]. With age, the number of inner ear hair cells of the vestibular system decreases [116]. Losses in proprioception, motion and position sense clearly influence sensorimotor tasks such as balance in the older adults [117].

In combination with losses in muscle force, velocity, and power, sensory degeneration will negatively influence human balance and posture. As a consequence, the number of falls almost doubles (195% between 35 and 76 yrs) with increasing age ([118], Fig. 3d). Females fall more often than males (215% vs. 175%), and the amount of people who report multiple falls per year increases with age [118]. The incidence for community-dwelling older adults is 0.7 falls per year [119]. As the number of falls almost doubles, and as the steps per day decrease by 75% (60 to 85 yrs, [87]), the falls per number of steps taken per day is approximately 800% higher for older adults compared to young adults.

The occurrence of fall injuries increases by 336% between the ages of 31 and 80, and larger increases were found up to the age of 90 years (up to 472%). Between 30% and 50% of older adult fallers become injured in a way that requires a doctor or to be limited in daily life activity for at least one day [120, 121]. Between the ages of 65 and 90 years, mortality rate increases from one to 40 deaths per 10,000 falls (4000%) [122]. Seventy-three percent of fall injuries occur during walking; 16% while walking on snow or ice, 45% while walking on other surfaces, and 12% while going up or down stairs [123]. Most falls (57%) were caused by slipping, tripping, or stumbling [124]. Other reasons for fall-related injuries are health problems (7%), from furniture or while rising from furniture (6%), sport (5%), and from elevated position (4%) [123]. Fall-related injuries have also been associated with a loss of balance, dizziness, fainting, or seizures (27%) [124].

An increased fall rate was reported for people with the respiratory disease COPD [125]. Further, an impact on balance was reported for people with CF [126], which may have been mainly due to reduced quadriceps strength. Increased rates of falling were also found for people with cardiovascular diseases such as PVD [127]). For people with the musculoskeletal disease FSHD, the yearly number of falls was four times higher compared to the unaffected control group [128]. For people with OA, the likelihood of falls was increased compared to controls, and was further increased with the number of affected lower limb joints [129]. Increased rates of falling were also reported for neurological diseases. Postural instability [130] and an increased rate of falling [131, 132] were reported for people with PD. Additionally, adults with CP experience reductions in mobility in early to middle adulthood in conjunction with reduced balance and increased risk of falling [133].

A combination of extrinsic (e.g. ground surface) and intrinsic reasons might be responsible for the increasing fall rates. Intrinsic reasons include identified losses in maximum muscle strength, power, reaction time, fatigue, or sensory losses.

Muscle strength was recommended to be assessed and treated in older adults to prevent falls [134]. Fall intervention studies showed a reduction of falls by 18% and 60% using muscle strength and balance training [132]. Ankle dorsiflexion weakness in particular seems to indicate risk of falling [135, 136]. Next to muscle weakness, fallers showed greater asymmetry in muscle force and muscle power between the lower limbs [135].

For rapid step testing it was demonstrated that younger subjects could recover from a larger body lean angle compared to older adults due to advantages in step velocity [104]. This indicates that high joint power, including torque and velocity, is required to minimize the time to recover from perturbations, such as stumbling or tripping. Increased reaction time, caused by sensory losses, may also increase perturbation recovery time.

Muscle fatigue may be an additional reason for increased fall rates in the older adults. Helbostad et al. [137] found no changes in self-selected gait speed or step length in a group of subjects with a mean age of 79 ±5 years after being fatigued by a sit-to-stand task. In contrast, subjects showed significant increases in step width and mediolateral trunk acceleration [137]. Increased step width was also identified when older adults (mean 61 ±6 yrs) were forced to walk at same speed as younger subjects (mean 25 ±3 yrs) [138]. When walking speed was not fixed, older adults preferred a similar step width, but lower walking speed, compared to young adults [80]. Researchers assume that walking speed might decrease to maintain balance [81] or to manage fear of falling [82].

#### Other identified conditions

In addition to the physical and functional changes analyzes in this review, we found other factors that may influence gait. One of the major issues addressed by multiple studies is pain. Compared to healthy individuals, physical disability (including walking) is five times higher for people with pain caused by OA [102]. Exercise and dietary weight loss can improve health related quality of life due to reductions in pain and physical disability [139–141]. Pain was also reported for people with the cardiovascular disease PVD where calf pain occurred due to deoxygenation during physical activity [101].

Another reported issue were asymmetries in gait. For example, increased asymmetries compared to the control group were found for people with PD and older adult fallers [142]. Additionally, next to asymmetries also groups with different walking patterns could be identified for people with CP [143].

For our analysis, most of the evaluated populations (Fig. 1) with mobility-related losses did have standing and walking capability. This included unimpaired older adults but also mobility-impaired individuals with respiratory, cardiovascular, neurological, and musculoskeletal diseases. One population with limited capability or without standing and walking capability were people with SCI [95].

Furthermore, freezing was reported to be a mobility limiting contributor for people with PD [144]. Following the definition of [144], freezing is defined as an episodic inability to generate effective stepping, mostly during turning and step initiation, but also when faced with stress or distraction. Focused attention and external stimuli can overcome the episode.

### Daily life requirements of physical parameters

#### Maximum muscle force and power

**Fig. 7:**
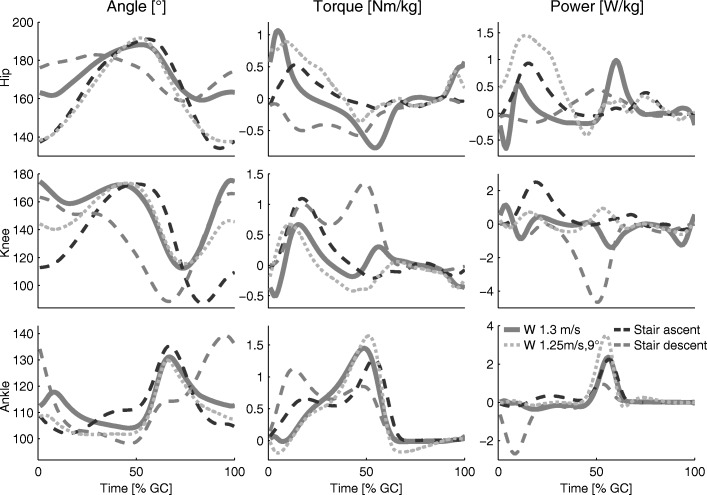
Joint biomechanics. Hip, knee, and ankle biomechanics (angle, torque, and power) for one gait cycle of level walking (solid, 1.3 m/s, [169]), walking inclines (dotted, 1.25 m/s, 9°, [170]), and ascending (dashed, black) and descending (dashed gray) stairs [148]. For [170] and [148], joint torques and angles were digitized. Joint angular velocity and power were calculated using these values in combination with the published gait cycle time information [171]

Compared to level walking, human peak power and torque from the ankle, knee, and hip increase with increasing slope [145–147]. The largest increases (compared to level walking) were identified for hip extension and plantarflexion torque and power (Fig. 7). Additionally, increased joint requirements could be identified while climbing stairs [148]. Compared to level walking, peak knee torque and power increases for ascending and descending stairs (Fig. 7).

#### Maximum oxygen consumption

Required oxygen consumption for 1.3 m/s level walking is 12 ml ·kg^−1^·min^−1^. An increased oxygen consumption has been found (18.4 ml ·kg^−1^·min^−1^) when increasing speed to 1.8 m/s (both values for unimpaired adults, mean 39 ±13 yrs, [149]). Compared to level walking, 1.3 m/s walking at a slope of 9° requires 28 ml ·kg^−1^·min^−1^ [150]. The human cost of transport, which quantifies the energy efficiency of gait, has been found to be 1.6 for level walking; for a slope of 6°, this cost tripled, and for a slope of 24°, this cost increased ten-fold (17.3) compared to that of level walking [151].

Similar to inclined walking, required oxygen consumption increases approximately three times (34 ml ·*kg*^−1^·*min*^−1^, 95 steps/min) for stair climbing compared to level walking ([152], 44 ±13 yrs). Approximately 30 ml ·*kg*^−1^·*min*^−1^ were required for a group of subjects with a mean age of 20 ±0.3 years (88 steps/min, [153]).

## Discussion

### Limitations due to physiological parameters

This review identified that lower limb maximum muscle torques and forces, as well as leg extensor power, decreased with increasing age. For daily movements, increased joint torque and power requirements were identified for walking inclines and climbing stairs compared to level walking (Fig. 7). Thus, it is expected that both movement tasks will most likely challenge older adults and mobility-impaired individuals. In [66], reduced quadriceps strength was identified as a reason for reduced stair climbing cadence in older adults. Additionally, older adults reached 75% of their maximum possible extensor moment in stair climbing, while younger adults reached 53% [67]. Thus, the effort of older adults is greater and muscle fatigue may occur earlier. We expect similar effects in user effort for level walking and walking inclines. Furthermore, limited muscle power is linked to incident disability, mortality, falls, hospitalization, and health care resource consumption [46].

This review identified a loss of VO_2_max with increasing age or due to diseases. As the oxygen consumption at self-selected walking speed is below the VO_2_max of most older adults (Fig. 4), these individuals should be able to handle the effort for short periods of time. With increasing locomotion time, sub-maximal values of VO_2_max must be considered. For intervals of three minutes, walking or running in the Bruce GXT test, values above 70% of VO_2_max were categorized as hard [154]. A study on carrying loads on different terrain in men and women showed that, for all different conditions, the self-selected pace of the subjects required 45% of the individual VO_2_max [155]. This value seems to be the acceptable working limit for a duration of one to two hours. For young soldiers carrying loads over six hours for multiple days, the self-selected pace was approximately at 30%-40% of the VO2max [156]. If these percentages of the VO_2_max are assumed as continuous limits for level walking, it might explain part of the reductions in maximum, maximum six-minute, and self-selected walking speed of older adults and those that are mobility-impaired. In addition to some percentile of older adults without observable limitations, in comparison to young adults there will be some percentile with great restrictions, similar to the distribution for the steps per day (Fig. 6). In comparison to level walking, oxygen requirements for stair climbing and walking inclines (with a speed of young adults) are above the maximum for most older adults (Fig. 4). To perform both tasks, older adults need to reduce their speed, similar to the strategy employed by mountain runners [151]. Studies of individuals with respiratory, cardiovascular, and neurological diseases showed clear reductions for VO_2_max to levels of less than the half of unimpaired subjects of the same age group (Fig. 4). In addition, maximum (six-minutes) and self-selected level walking speed of the impaired populations analyzed were below the mean self-selected level walking speed of the unimpaired controls (Fig. 5). Thus, these groups are likely to struggle to perform daily locomotion tasks at self-selected speeds compared to unimpaired individuals of the same age.

Older adults showed only small reductions in self-selected walking speed compared to the reductions in maximum muscle force, maximum power, and VO_2_max. Thus, maximum physiological parameters seem to impact maximum performance (e.g. maximum walking speed) to a greater degree than movements that only require medium level effort (e.g. preferred walking speed). Typically daily locomotion is done at speeds up to the self-selected walking speed, which should require a medium level effort. But the number of steps per day decreased much more with increasing age than the physiological values (e.g. force, VO_2_max). This suggests that not only physiological, but other factors, such as not having a need to work, might play an important role in the reduction in steps per day.

### Improving the functional capacity

Based on the physical and functional parameters analyzed in this work, we identified several mobility-related losses, due to aging and diseases, that have the potential to be improved. Functional improvements can include upright standing and locomotion, increasing locomotion speed, steps per day, reaction time, improving balance (risk of falling), or improving gait patterns, which includes the reduction of asymmetries.

We found that most functional tasks are affected by the same physical deficits, including muscle strength, muscle power, and VO_2_max. Consequently, with reduced levels, other factors such as fatigue, effort, pain, or joint stress have the potential to increase.

As physical and functional parameters are highly related to each other, it is not surprising that losses due to aging or disease in one area also reduce capabilities in other areas. For example, individuals with cardiovascular diseases (PVD) suffer from increased reaction times and fall rates, or individuals with respiratory diseases (COPD) suffer from reductions in maximum muscle power. Thus we believe that improvements in the physical capabilities have the potential to improve a wide range of functional parameters.

The authors see two possible options to improve mobility-related functional parameters (e.g. steps per day), and consequently, secondary parameters as well (e.g. pressure sores, body mass index).

The first potential solution is physical training, as physical inactivity was identified as a major cause for physical losses. Training directly targets the improvement of a specific capacity and can partially prevent or help to recover from physical losses.

The second potential solution would bypass the human physical losses to directly improve the mobility by improving the functional capacity. Next to the training approach, this approach is required as this review identified that there will be an inevitable loss of capabilities, especially for older adults from the age of above 70 yrs and for mobility-impaired individuals.

Until now, changes in the environment or the use of assistive devices, such as crutches or walkers, have been used and investigated to compensate for inevitable losses in physical and functional capabilities. Alternatively, assistive devices can also be used during rehabilitation as training devices.

A novel assistive device concept that can address these two options for functional improvements are exoskeletons. Similar to crutches, exoskeletons can be used for daily assistance (compensation) and as a rehabilitation device (recovery). In addition to the improvement of the physical condition, improvements of secondary medical symptoms as well as other movement- and posture-related health outcomes are expected. These improvements will be beneficial for the users also when not wearing the exoskeleton. Compared to devices like crutches, they could also be used as a versatile training device to partially prevent losses similar to other physical exercise devices [157]. In addition to prevention, the functional compensation, and rehabilitation from losses, exoskeletons provide the possibility to augment user capabilities to levels above that of normal human performance. For example, when using the Raytheon Sarcos’s XOS 2 robotic suit, the user should be able to lift 200 lb of weight for long periods of time without feeling the strain [158]. So far it is unknown how different levels of assistance will influence the physical capabilities of the users. To prevent from further physical losses, the trade-off between exoskeleton assistance and physical user involvement has to be investigated. We can imagine that muscles might degenerate if the user completely relies on the external force assistance of an exoskeleton. On the other hand, too much effort may overload and fatigue the user. Variable assistance levels, controlled by parameters that indicate human effort (e.g. heart rate) might be a possible way to set an appropriate level of effort.

Thus far commercial exoskeletons have been primarily used in rehabilitation [159]. A review on lower limb rehabilitation exoskeletons concluded that exoskeletons can be used to regain locomotion capability for impaired with neurological diseases. They can increase mobility, improve functioning, and reduce the risk of secondary injury by reinstating a more normal gait pattern [159]. For the devices investigated in this review (most commonly ReWalk, HAL, Vanderbilt lower limb exoskeleton), user’s mobility benefited from the exoskeletons body weight support and the propulsion during walking.

Needs such as the compensation for lost locomotion speed or endurance and the reduction of fatigue and effort, may require exoskeletons, which are able to reduce the metabolic cost of walking by providing propulsion to the lower limbs. Examples for autonomous designs that are able to reduce metabolic cost of walking by assisting the hip are from Samsung [33], Honda [34], or Georgia Tech [35]. An autonomous systems with ankle support was designed by MIT [32]. Ankle and hip assistance was provided with the exosuit from Harvard [16].

A reduction of gait asymmetries could potentially be addressed with unilateral systems like the ankle exosuit [160, 161], or with bilateral systems similar to the Ekso-GT [162], which has demonstrated improved gait metrics by providing propulsion at the deficient limb of people with stroke.

The risk of falling may be reduced by reducing fatigue and asymmetries, improving strength and power, or by using control algorithms within exoskeletons or assistive devices that improve balance or assist to recover from perturbations, as demonstrated in [163]. As increased reaction times have been associated with falls [164], artificial sensors in combination with assistive forces could also help to compensate for the human sensory losses.

To reduce joint stress and pain, exoskeletons have to reduce the forces on the cartilage and the bones. Increasing joint stability by antagonistic structures may further decrease pain while moving.

While there are many of gait rehabilitation exoskeletons for clinical environments, there are only a few exoskeletons available that are solutions for improving mobility in daily life for many of the mobility impairments discussed in this work. Necessary technological advances that will allow for greater widespread daily use include improvements to the actuators, sensors, batteries, and the human machine interface. Furthermore, it must be investigated how the control of such assistive devices can deal with different gait patterns, as found in individuals with diseases such as CP [143]. Next to individual solutions, people with CP, PD, and other diseases require solutions to deal with symptoms like tremors, spasticity, and involuntary movements.

While we see a huge potential to improve the mobility of individuals with the help of lower limb exoskeletons, we believe there is still a lot of development required to create systems that fulfill the needs for the different populations with reduced mobility. Hardware and control complexity should be user-friendly and cover the needs of the desired target population.

### Questioning the necessity of lower limb exoskeletons

It is hard to estimate, which level of fatigue, effort, pain, or fall risk would make individuals to choose to use an exoskeleton for daily life mobility assistance. Conventional training, medication, passive walkers or crutches, or even a reduction in movement speed may be preferred alternatives. For shorter distances in level environments in particular, a high amount of older adults without severe physical and functional deficits will not require a lower limb exoskeleton for assistance. The possible benefits of reduced effort or risk of falling might be rated lower compared to the effort of donning and doffing or charging of the exoskeleton. Further, financial expenses for the device could be disincentive for use.

To establish the usage, the advantages of exoskeletons must be perceived to be higher by the users compared to the disadvantages. We clearly see this for target populations with severe mobility impairments due to diseases. On the other hand, we could imagine that also young and healthy people might use such devices to augment their capabilities at the workplace or for activities such as hiking or running. User-friendly (e.g., robust, simple) exoskeleton solutions that work for these applications might also improve the accessibility for populations with moderate limitations in mobility.

## Conclusions

Mobility is a key determinant for individual independence and quality of life. This review summarized and quantified mobility related physical and functional losses with increasing age and due to diseases.

We found decreases in maximum walking speed (24%, 25–75 yrs), maximum six-minute walking speed (38%, 20–85 yrs), and self-selected walking speed (21%, 20–85 yrs). Between the ages of 25 and 75 years, lower extremity maximum muscle strength decreases by 33%, VO_2_max decreased by 40% and muscle power decreased by 49%. Single reaction time can more than double and complex reaction time can almost triple (25 to 90 yrs). In addition, the balance related visual system, the vestibular system, and the somatosensory system degenerate with increasing age. Steps per day decrease by 75% (60 to 85 yrs). The falls per number of steps taken per day increase by 800% and injuries due to falls are almost five times greater when comparing young adults to older adults at the age of 90. The mortality rate due to falls increases by 4000% when comparing 65 year old to 90 year old subjects.

This review demonstrates that increasing age and diseases reduce mobility related capabilities for a broad range of populations. For shorter walking distances in level environments, most older adults will be able to remain mobile with a reduced walking speed. In contrast, we found large populations with severe mobility impairments who may struggle, especially in demanding tasks such as walking inclines, climbing stairs, or walking over longer periods of time. As a result of these tasks being close to their physiological limits, both fatigue as well as falls may increase. Other identified contributing factors to losses in mobility were the losses in the ability to stand and walk, physical and functional asymmetries, breathlessness, fear of falling, deoxygenation with calf pain, joint stress and pain, and freezing. Further, this study revealed much larger populations with mobility impairments in walking capability compared to populations without. Thus, we see an increased need for mobility enhancing solutions for impaired populations that have partial, and not necessarily total, mobility limitations.

As this review showed that physical and functional parameters are closely related to each other, we believe that improvements in the physical parameters can improve a wide range of functional and secondary measures. Directly targeting the prevention of physical losses and the improvement of physical capabilities by training is one attractive approach to improve mobility. On the other hand, there are inevitable physical losses with increasing age or due to mobility impairments. Solutions are required to compensate for these losses, such as with environmental changes or assistive devices.

We believe that exoskeletons are a promising assistive device that can be used for training to prevent or recover physical losses. These devices allow for the compensation of lost physical capabilities by directly supporting the functional tasks with propulsion, weight support, or balance support. Thus, they have the potential to increase a user’s functional capacity to levels that equal unimpaired young individuals or to augment functional capabilities to levels beyond natural human capabilities.

Future studies are necessary to explore the potential for exoskeletons to address the physical and functional losses at various levels (prevention, recovery, compensation, augmentation). It will be of interest to understand how exoskeletons will affect secondary medical symptoms as well as other movement- and posture-related health outcomes. We expect improvements in other health-related measures, and therefore also improvements in quality of life when not wearing the assistive device. To establish the usage of exoskeletons, devices must be user-friendly and the mobility advantages must be perceived to be greater by the user compared to the associated disadvantages.

## Appendix

### Methodological considerations

This narrative review used selected articles to provide an overall view on the physical and functional losses due to aging and diseases. The summarized losses that were extracted from literature only represent the investigated population of the original work. It is possible that populations with other characteristics (e.g., income, education, ethnicity, sex, age) may have different losses. Studies used as example for impaired populations were only single study representatives. Groups with more severe or more moderate disease symptoms may have greater or lower losses.

Further, we can not exclude that subjects that were characterized as healthy or controls without the investigated disease, might have suffered from diseases that were not used as exclusion criteria in the study protocols. Thus, group or study comparisons may have been influenced due to other diseases causing similar physical or functional changes.

Another point that might not have changed the general outcome, but the relative relation, are the comparisons of different age ranges. For example, for the maximum walking speed, a range of 25 to 75 years was used and for the self-selected speed a range of 20 to 85 years was used. We preferred to include the full range instead of truncating the age groups above 75 years, as the greatest changes were expected within this group.

**Table 2:** Comfortable walking speed (self-selected) at different ages for healthy males (M), females (F), and mixed populations (M & F)

Study	Gender	Age range	Mean age	Mean speed	Setting	Number of subjects
Murray [182]	M	20 - 25	21.5	1.50	10m walkway	8
	M	30 - 35	32.4	1.43		8
	M	40 - 45	42.9	1.59		8
	M	50 - 55	53.0	1.57		8
	M	60 - 65	62.8	1.45		8
	M	67 - 73	71.1	1.18		8
	M	74 - 80	76.0	1.23		8
	M	81 - 87	85.0	1.18		8
Hageman [183]	F	20 - 33	23.9	1.60	10m walkway	13
	F	60 - 84	66.6	1.32		13
Waters [149]	F	20 - 59	40.1	1.29	60.5m walkway	34
	F	60 - 80	68.9	1.20		47
	M	20 - 59	38.5	1.36		39
	M	60 - 80	67.1	1.28		26
	M & F	20 - 59	39.2	1.33		73
	M & F	60 - 80	68.2	1.23		73
Blanke [184]	M	20 - 33	24.5	1.31	14m walkway	12
	M	60 - 74	63.6	1.39		12
Elble [80]	M & F	20 - 39	30.0	1.18	10m walkway	20
	M & F	65 - 87	74.7	0.94		20
Öberg [185]	M	20 - 29	24.5*	1.23	10m walkway	15
	M	30 - 39	34.5*	1.32		15
	M	40 - 49	44.5*	1.33		15
	M	50 - 59	54.5*	1.25		15
	M	60 - 69	64.5*	1.28		15
	M	70 - 79	74.5*	1.18		14
	F	20 - 29	24.5*	1.24		15
	F	30 - 39	34.5*	1.29		15
	F	40 - 49	44.5*	1.25		15
	F	50 - 59	54.5*	1.11		15
	F	60 - 69	64.5*	1.16		15
	F	70 - 79	74.5*	1.11		15
Ostrosky [186]	M & F	22 - 39	28.2	1.38	6m walkway	30
	M & F	60 - 80	67.5	1.27		30
Bohannon [187]	M	50 - 79	64.4	1.41	7.6m walkway	77
	F	50 - 79	64.3	1.30		79
Bohannon [63]	M	20 - 29	23.9	1.39	7.6m walkway	15
	M	30 - 39	34.2	1.46		13
	M	40 - 49	44.9	1.46		22
	M	50 - 59	54.9	1.39		22
	M	60 - 69	66.2	1.36		18
	M	70 - 79	73.0	1.33		22
	F	20 - 29	22.2	1.41		22
	F	30 - 39	35.1	1.42		23
	F	40 - 49	44.1	1.39		21
	F	50 - 59	53.8	1.40		21
	F	60 - 69	64.8	1.30		18
	F	70 - 79	73.1	1.27		20
Auvinet [188]	M	20 - 29	24.5*	1.59	40m walkway	24
	M	30 - 39	34.5*	1.54		26
	M	40 - 49	44.5*	1.63		22
	M	50 - 59	54.5*	1.42		25
	M	60 - 69	64.5*	1.47		28
	M	>70	74.5*	1.32		13
	F	20 - 29	24.5*	1.54		25
	F	30 - 39	34.5*	1.56		27
	F	40 - 49	44.5*	1.50		29
	F	50 - 59	54.5*	1.48		24
	F	60 - 69	64.5*	1.35		25
	F	>70	74.5*	1.26		14
Malatesta [189]	M & F	62 - 70	66.9	1.38	treadmill	10
	M & F	79 - 87	82.8	1.16		10
Menz [190]	M & F	22 - 40	28.5	1.43	8.6m walkway	30
	M & F	76 - 87	80.8	1.16		31
Laufer [191]	M & F	20 - 31	24.0	1.46	6.6m walkway	30
	M & F	65 - 89	77.7	1.00		40
Kang [192]	M & F	18 - 28	23.3	1.30	treadmill	18
	M & F	65 - 85	72.1	1.29		18
Mazza [193]	M & F		24.4	1.30	12m walkway	16
	M & F		72.0	0.97		20
Kavanagh [194]	M & F		23.0	1.32	30m walkway	13
Mazza [195]	M		23.0	1.33	12m walkway	20
	F		23.0	1.34		20
Chung [196]	M	<30	27.4	1.08	8m walkway	5
	M	31 - 45	39.8	1.12		5
	M	>45	51.0	0.99		5
	F	<30	23.6	1.20		5
	F	31 - 45	31.2	1.11		5
	F	<30	57.6	0.90		5
Iosa [71]	M & F		31.0	1.21	10m walkway	13
Goutier [197]	M		22.0	1.30	12.5m walkway	10
	M		71.0	1.30		10
	F		24.0	1.30		10
	F		71.0	1.20		10
Peterson [198]	M	20 - 30	24.7	1.28	12.5m walkway	6
	M	65 - 80	72.7	1.31		8
	F	20 - 30	26.4	1.38		6
	F	65 - 80	69.9	1.32		8
Lamoth [199]	M & F	>70	79.4	0.95	40m walkway	13
Ijmker [200]	M & F	55 - 70	64.3	1.19	10m walkway	12
	M & F	75 - 85	76.9	1.14		14
Iosa [167]	M & F		62.8	1.15	20m walkway	10
Senden [201]	M & F		74.2	1.23	40m walkway	50
Arnold [202]	M & F		23.2	1.34	10m walkway	20
	M & F		73.2	1.14		20
Terrier [203]	M & F	20 - 29	24.7	1.10	treadmill	20
	M & F	30 - 39	34.6	1.13		20
	M & F	40 - 49	43.9	1.11		20
	M & F	50 - 59	54.8	1.04		20
	M & F	60 - 69	63.3	1.06		20

Depending on the study, speed was determined at a walkway or a treadmill. For some studies the age range was not specified. If the mean age was not published, the mean of the age range was used (*). Self-selected speed was determined in some studies using short walkways. It is unclear how representative such a measurement is for self-selected level walking speeds over longer periods of time. Slower values are expected

For some age-related parameters (e.g. VO_2_max, power, see Fig. 3), functions were introduced by the referenced authors, based on a linear fit. These linear trends might hide non-linear effects that we would have expected with increasing age.

### Diseases information

#### Respiratory system

The WHO estimated 64 million cases worldwide of chronic obstructive pulmonary disease (COPD) for the year 2004 [172]. Due to chronically reduced airflow, people with COPD show reduced activity during the day compared to healthy older adults [100].

Cystic fibrosis (CF, mucoviscidosis) is a metabolic disease caused by a genetic defect. The incidence is increased within the Caucasian population. One in 2000 to 3000 new-borns are affected in Europe, and one out of 3500 in the US [174]. The incidence is much lower in Asia and Africa. 30,000 cases are registered in the US [179]. Combining the values for the US with an estimation of cases for Europe, more than 100,000 people are affected.

#### Cardiovascular system

For coronary artery disease (CAD), plaque at the inner site of the heart arteries causes a reduced blood flow and therefore a reduced supply of the heart. It is the leading cause of death worldwide (7.2 million per year, WHO, [172]). Worldwide 93 million are affected [173].

Peripheral vascular disease (PVD) describes the reduction in blood flow in the extremities mostly caused by arteriosclerosis. Worldwide, a number of 202 million was estimated to be affected in 2010 [175].

#### Musculoskeletal system

Osteoarthritis (OA) is a degenerative disease of the bones and cartilage at the joints. 151 million people are affected worldwide [172].

Facioscapulohumeral muscular dystrophy (FSHD) is a genetic disease that causes muscle atrophy and weakness. Based on the prevalence of 1 in 8333 inhabitants in the Netherlands [176], the FSH SOCIETY [180] estimates 870,000 affected worldwide.

#### Neurological system

Cerebral palsy (CP) describes functional disability of movement and/or posture caused by an abnormally developed brain [181]. It affects 2 to 2.5 per 1000 live births [177]. Assuming a similar life expectancy to non-affected and a similar incidence for all countries, 16 million people may be affected worldwide.

Parkinson’s disease (PD) is a degenerative nerve disease caused by dying midbrain cells. It is estimated to affect 5.2 million people worldwide [172].

Spinal cord injury (SCI) has a prevalence of 223–755 per million inhabitants [178], which totals about 3.5 million cases worldwide. It is primarily caused by traumatic injuries, but it has also non-traumatic causes (arthritis, reduced blood flow, infection, inflammation).
