# Correction to: Mobility related physical and functional losses due to aging and disease - a motivation for lower limb exoskeletons

**DOI:** 10.1186/s12984-020-0648-z

**Published:** 2020-02-19

**Authors:** Martin Grimmer, Robert Riener, Conor James Walsh, Andre Seyfarth

**Affiliations:** 1 Lauflabor Locomotion Lab, Technische Universität Darmstadt, Magdalenenstr. 27, 64289 Darmstadt, Germany; 2 Department of Health Sciences and Technology, Sensory-Motor Systems (SMS) Lab, Institute of Robotics and Intelligent Systems (IRIS), ETH Zurich, Tannenstr. 1, 8092 Zurich, Switzerland; 3 Harvard Biodesign Lab, John A. Paulson School of Engineering and Applied Sciences, Wyss Institute for Biologically Inspired Engineering, Harvard University, 60 Oxford Street, Cambridge, MA 02138 USA

**Correction to: J Neuroeng Rehabil**

**https://doi.org/10.1186/s12984-018-0458**

The original article [1] contains an error in Fig. 3f whereby data is erroneously extrapolated beyond 80 years of age; this also affects statements made elsewhere in the article.

**Fig. 3:**
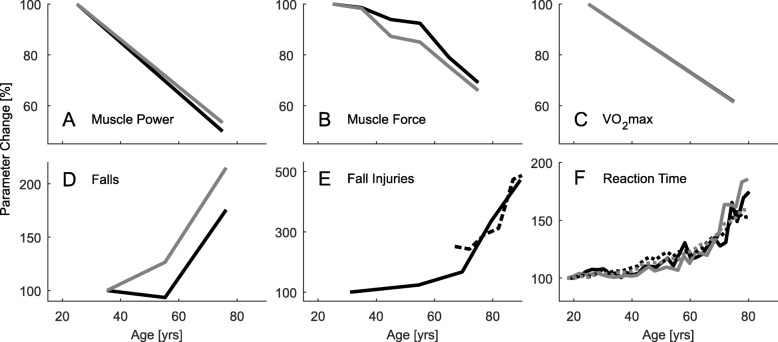
Summary of age related parameters. Changes with age in maximum muscle power (**a**), maximum muscle force (**b**), maximum oxygen consumption (**c**), self reported falls (**d**), injuries due to falls (**e**), and reaction time (**f**). Black lines represent male, gray lines female and dashed lines mixed groups. **a** Muscle power data was assessed by jumping mechanography (89 male, 169 female, 18-88 yrs) [46]. **b** Muscle force data is the mean of the curves presented in Fig. 2. **c** Maximum oxygen consumption was assessed in treadmill walking from (619 male, 497 female, 18-94 yrs) [54]. The relation of VO_2_max and age is described as *y*=51.23−0.33·*x* for males and *y*=41.74−0.27·*x* for females. **d** Changes in self reported falls (one minimum in the last two years) for three age groups in percent. Age means were 35.3 (20–45, *n*=292), 55.3 (46–65, *n*=616), and 76.2 (>65, *n*=589) years. The relative amount of male fallers is 16.8, 15.7, and 29.5 percent and of female fallers is 20, 25.3, and 43 percent with increasing age [118]. **e** Increases of injuries due to falls (survey, 30–90 yrs) for the Canadian (dashed, [123]) and the US (solid, [124]) population with 100% set for 30 years old of [124]. Absolute values are about 20 to 100 falls with injury per 1000 population for the 30 and 90 years old respectively. **f** Relative change with age (100% at 18 yrs) of single (dotted) and choice (solid) reaction time of 7130 subjects (18-90 yrs, [103]). Absolute values range from 287 ms to 872 ms for the single and 567 ms to 1129 ms for the choice reaction. Data was acquired using a single button that had to be pressed when showing a number in a display. Choice reaction time included pressing one out of four different buttons

Thus, the correct version of Fig. 3f can be viewed ahead and should be considered in place of the original Fig. 3f; furthermore, the following amendments to affected statements should also be considered:

1. **Abstract**

- Original article statement:
  “Reaction times more than double (18–90 yrs)”
- Corrected statement:
  “Reaction times can almost double (18-80 yrs)”

2) **Results**

- Original article statement:
  “While single reaction time can more than double, choice reaction time can almost triple with increasing age (25 to 90 yrs, Fig. 3f, [103]).”
- Corrected statement:
  “While single reaction time increases to 180%, choice reaction time increases to 160% with increasing age (18 to 80 yrs, Fig. 3f, [103]).”

3) **Conclusion**

- Original article statement:
  “Single reaction time can more than double and complex reaction time can almost triple (25 to 90 yrs).”
- Corrected statement:
  “Single reaction time increases to 180%, choice reaction time increases to 160% (18 to 80 yrs).”

## References

[1] Grimmer M, Riener R, Walsh CJ, Seyfarth A (2019). Mobility related physical and functional losses due to aging and disease - a motivation for lower limb exoskeletons. J Neuroeng Rehabil.

